# Perceptions of Neighborhood Safety and Asthma among Children and Adolescents in Los Angeles: A Multilevel Analysis

**DOI:** 10.1371/journal.pone.0087524

**Published:** 2014-01-23

**Authors:** Marlene Camacho-Rivera, Ichiro Kawachi, Gary G. Bennett, S. V. Subramanian

**Affiliations:** 1 Department of Population Health, North Shore-LIJ Health System, Great Neck, New York, United States of America; 2 Department of Social and Behavioral Sciences, Harvard School of Public Health, Boston, Massachusetts, United States of America; 3 Department of Psychology and Neuroscience, Duke University, Durham, North Carolina, United States of America; Universidade Federal do Acre (Federal University of Acre), Brazil

## Abstract

**Background:**

Research examining the impact of neighborhoods on asthma has shown an increased interest in the role of the psychosocial environment. We examined the associations between various measures of neighborhood safety, individual and family characteristics, and asthma outcomes among children in Los Angeles.

**Methods:**

Multilevel logistic regression models were used to analyze data on 3,114 children across 65 neighborhoods from Wave 1 of the Los Angeles Family and Neighborhood Survey (2000 to 2002). Primary caregivers reported asthma outcome and all individual covariates; home environmental characteristics were observed by the interviewer.

**Results:**

In fully adjusted models, parents who reported their neighborhood fairly safe or somewhat dangerous had lower odds of reported lifetime asthma compared to those who reported their neighborhood completely safe (OR 0.71; 95% CI 0.52–0.96 and OR 0.60; 95% CI 0.42–0.88 respectively). Conversely, parents who reported they could not trust their neighbors to keep their children safe had a nearly 40% increase in lifetime asthma compared to those who reported they could trust their neighbors to keep their children safe (OR 1.39; 95% CI 1.07–1.81).

**Conclusions:**

The study demonstrates a complex pattern between various measures of neighborhood safety and asthma and suggests that these relationships may operate differently in Los Angeles. As an increasing proportion of children are growing up in newer Western and Southwestern cities, which have different physical layouts and residential segregation patterns compared to Northeast and Midwestern cities, future studies should continue to examine neighborhood psychosocial stressors and asthma in diverse contexts.

## Introduction

While prior research examining the impact of neighborhoods on asthma has primarily focused on environmental exposures and the physical environment such as housing conditions there has been increasing interest in the role of the social environment.[1]–[5] Researchers have taken an increased interest in the role of neighborhood violence and have linked exposure to neighborhood violence to asthma onset, hospital emergency room visits, and symptoms. [6], [7] The Inner-City Asthma Study demonstrated an association between higher levels of perceived neighborhood violence and increased caretaker-reported asthma symptoms among children ages 5 to 12 years. [7] Additional aspects of neighborhood violence, such as measures of crime and presence of gangs, have also been associated with childhood asthma symptoms and hospital visits. [8], [9] Retrospective studies have also found lifetime exposure to neighborhood violence to be associated with an increased risk of asthma and wheezing. [10] A longitudinal study of children living in Chicago neighborhoods found a robust positive association between exposure to community violence and risk of asthma development [11].

Much of the research linking exposure to neighborhood violence and asthma stems from the work of Wright and colleagues, who hypothesize that exposure to violence may affect asthma through direct and indirect pathways. [10] Directly, exposure to violence may increase psychological stress experienced by those who witness or are victims of violence.[12]–[14] Numerous studies have pointed to the link between over-activation of immune-inflammatory systems and increased susceptibility to respiratory illnesses. [15], [16] However, some studies that have examined the association between neighborhood factors and asthma have found associations through behavioral pathways [9].

Indirect pathways may include increased exposure to indoor allergens or adverse parental behaviors. The association between exposure to indoor allergens, such as dust, cockroach, and mold and asthma related outcomes has been well documented. [17], [18] Parental report of keeping their children indoors because of fear of neighborhood violence was related to increased risk of wheeze and asthma among children living in inner-city Boston. [7] High violence rates and other adverse life events may influence parents’ behaviors, including missing medical visits, failing to obtain medications, or smoking. [19] At the family level, there is strong evidence that family conflict, parenting difficulties, and parental stress are associated with wheezing in infancy, asthma onset and hospitalizations. [7], [20]–[22].

While studies have shown associations between neighborhood social stressors and childhood asthma, only two studies have explicitly examined perceptions of neighborhood safety and asthma. Prior studies have been unable to control for features of the home environment as well as other neighborhood characteristics associated with asthma, such as neighborhood poverty. [23],[24] We investigated the association between various measures of neighborhood safety perception and reported lifetime asthma using data from Wave 1 of the Los Angeles Family and Neighborhood Survey (L.A.FANS). We examined whether primary caregiver’s perception of neighborhood safety is associated with asthma morbidity in children, controlling for individual and family characteristics and whether experience of crime within the neighborhood is associated with asthma morbidity in children, controlling for individual and family characteristics.

## Methods

### Subjects: Los Angeles Family and Neighborhood Survey (L.A.FANS)

Wave 1 L.A.FANS participants included a representative cross-sectional sample of all households across 65 neighborhoods (census tracts) of in Los Angeles County; poor neighborhoods and families with children were oversampled. [25], [26] Participant interviews were completed between April 2000 and January 2002, with high completion rates among respondents selected for interview, 89% of primary care givers, 87% of randomly selected children, and 86% of siblings of randomly selected children. Interviews were successfully completed for 3200 children and adolescents (ages 0–17 years), with a balanced distribution across age groups.

Wave 1 participants provided data on individual, familial, and neighborhood factors, for themselves and household members; census data was also incorporated to provide social characteristics of each neighborhood, such as poverty levels and racial/ethnic composition. Our analyses included 3,114 children and adolescents (ages 0–17 years) with data on our outcome of interest.

### Outcome Measure

We ascertained child’s lifetime asthma status via the primary caregiver’s report of an asthma diagnosis within the parent module of L.A.FANS. Children were categorized as asthmatic if the primary caregivers reported a positive response to the following item: “has a doctor or other health professional ever told you that [child’s name] has asthma?” Similar questions to ascertain asthma diagnosis have been used within the International Study of Asthma and Allergies in Childhood (ISAAC) survey and the National Survey of Children’s Health [27], [28] Over 97% of children within L.A.FANS had a response to this question.

### Exposure Measures

Perceived neighborhood safety was measured by several items administered in the adult module of L.A.FANS. Experiences of neighborhood crime were assessed via response to the following item, “while you have lived in this neighborhood, have you or anyone in your household had anything stolen or damaged inside or outside your home, including your cars or vehicles parked on the street?” An additional measure of perceived neighborhood safety was captured through the adult’s extent of agreement with the following item, “you can count on adults in this neighborhood to watch out that children are safe and do not get in trouble”; response options for this item were strongly agree, agree, unsure, disagree, and strongly disagree. Participants were also asked how safe is it to walk around alone after dark within your neighborhood, with response options as extremely safe, somewhat safe, somewhat dangerous, or extremely dangerous.

### Covariates

At the individual level, child’s age, gender, and race/ethnicity were included. At the household level, we examined primary caregiver’s education (years), primary caregiver’s history of asthma, and primary caregiver’s current smoking status, health insurance status, and use of public assistance within the past 12 months. Covariates controlling for the home environment include whether the interviewer observed the presence of crowding, cleanliness or clutter, and potential health or structural hazards inside and immediately outside of the home at the time of the interview.

### Statistical Methods

To examine associations between asthma outcomes and perceived neighborhood safety, individual, and family characteristics, we conducted a series of two level multilevel logistic regression models of 3,114 children at level 1 nested within 65 census tracts at level 2. Use of multilevel modeling allows us to account for natural and sampling induced nesting within L.A.FANS, as well as model contextual heterogeneity; directing inquiry away from average effects, to inquire about differences and examine potential neighborhood variation in asthma. [29] Multilevel models are also appropriate when causal processes are thought to operate at more than one level; as asthma is a multi-factorial disease which is influenced not only by compositional factors (such as genetics) but also by contextual factors (such as neighborhood violence), single level regression models would be inappropriate. [30] We first examined the effects of neighborhood safety characteristics on the odds ratio of reporting an asthma diagnosis (Model 1) and subsequently adjusted for the effects of individual characteristics (Model 2), followed by primary caregiver’s characteristics (Model 3), and lastly physical characteristics of the indoor home environment and neighborhood poverty (Model 4). Quasi-likelihood methods were used to estimate the coefficients beginning with marginal quasi-likelihood (MQL) with 1^st^ order Taylor linearization to obtain starting values for 2^nd^ order penalized quasi-likelihood (PQL) approximation. Data manipulation and descriptive analyses were conducted using STATA 11, while multilevel models were conducted using MLwiN version 2.10.

### Ethics Statement

The data were collected by the RAND Corporation in collaboration with the UCLA School of Public Health. Written consent for participation in the study was obtained for L.A.FANS respondents by RAND Corporation in collaboration with the UCLA School of Public Health. Data for secondary analyses were obtained through submission of a restricted application process which included a data safeguarding plan, data user agreement, and IRB review. The research was approved by the Harvard School of Public Health Office of Human Research Administration.

## Results

Of the 3,114 children and adolescents (ages 0–17) in Wave 1 of L.A.FANS with data on the asthma outcome, 345 children (11%) had ever received a physician diagnosis of asthma. Table 1 displays the sample characteristics and 171 unadjusted associations between asthma and each of the individual, family, and household characteristics. At the individual level, the odds ratio (OR) of reporting a lifetime asthma diagnosis was lower among girls compared to boys and increased with age. Racial/ethnic differences were also observed, with non-Hispanic Black children having an over 2.5 fold increase in reporting a lifetime diagnosis for asthma compared to non-Hispanic White children; no other statistically significant racial/ethnic differences were observed. Primary caregiver’s history of asthma was significantly associated with reporting a lifetime asthma diagnosis, while primary caregiver’s current smoking status was marginally significant. Upon examination of primary caregiver’s education and public assistance, odds ratio of reporting a lifetime diagnosis of asthma increased with levels of education and were higher among those with health insurance. Only visible lack of cleanliness in the home environment was associated with an increased likelihood of reporting an asthma diagnosis.

**Table 1:** Number (percent) of participants by individual, family, and neighborhood factors among asthmatics and non-asthmatics (*n* = 3114).

	Asthmatics (n = 345)	Non-asthmatics (n = 2769)	Crude OR (95%CI)
*Individual characteristics*			
**Gender**			
Female	131 (38.0)	1385 (50.0)	0.73 (0.58–0.91)*
Male	214 (62.0)	1384 (50.0)	1.00
**Age (years)**			
≤5	79 (24.6)	1021 (36.6)	1.00
6–10	102 (29.6)	840 (30.5)	1.46 (1.08–1.97)*
11–14	97 (28.1)	536 (19.3)	2.11 (1.55–2.88)*
≥15	67 (17.7)	372 (13.6)	1.89 (1.33–2.69)*
**Race/ethnicity**			
Non-Hispanic White	63 (18.3)	503 (18.2)	1.00
Hispanic	182 (52.7)	1809 (65.3)	0.89 (0.64–1.26)
Non-Hispanic Black	62 (18.0)	221 (8.0)	2.59 (1.71–3.93)*
Asian/other	38 (11.0)	236 (8.5)	1.44 (0.93–2.24)
*Family characteristics*			
**Primary caregiver’s asthma history**			
No prior asthma diagnosis	270 (82.8)	2406 (94.2)	1.00
Prior asthma diagnosis	56 (17.2)	147 (5.8)	3.34 (2.38–4.67)*
**Primary caregiver’s smoking status**			
Non-smoker	56 (17.2)	336 (13.2)	1.00
Smoker	270 (82.8)	2217 (86.8)	1.37 (1.00–1.87)
**Health insurance during past 12 months**			
Insured	252 (76.8)	1726 (65.4)	1.00
Uninsured or Partially Insured	76 (23.2)	913 (34.6)	0.58 (0.44–0.75)*
**Primary caregiver’s education (years)**			
<12	92 (26.7)	1172 (42.3)	1.00
12	73 (21.2)	492 (17.8)	1.84 (1.33–2.54)*
>12	176 (51.0)	1080 (39.0)	2.02 (1.53–2.66)*
**Environment outside home unsafe for children**			
Yes	42 (12.3)	298 (10.9)	1.18 (0.83–1.68)
No	291 (84.8)	2372 (86.4)	1.00
**Environment inside home unsafe for children**			
Yes	17 (5.2)	146 (5.5)	0.96 (0.57–1.60)
No	311 (94.8)	2498 (94.5)	1.00
**Inside of home is crowded**			
Yes	52 (16.0)	382 (14.6)	1.15 (0.78–1.70)
No	272 (84.0)	2233 (85.4)	1.00
**Visible rooms are neat and uncluttered**			
Yes	199 (62.6)	1662 (66.3)	1.00
No	119 (37.4)	845 (33.7)	1.20 (0.95–1.53)
**Visible rooms are clean**			
Yes	280 (87.0)	2319 (91.3)	1.00
No	42 (13.0)	220 (8.7)	1.66 (1.17–2.36)*
*Neighborhood characteristics*			
**How safe to walk alone after dark in this neighborhood**			
Completely safe	76 (23.4)	467 (18.2)	1.00
Fairly safe	150 (46.2)	1221 (47.5)	0.71 (0.53–0.97)*
Somewhat dangerous	80 (24.6)	755 (29.4)	0.58 (0.41–0.84)*
Extremely dangerous	39 (11.3)	326 (11.8)	0.77 (0.44–1.37)
**Adults watch out that kids are safe**			
Strongly agree or agree	222 (67.7)	1875 (72.6)	1.00
Unsure, disagree, or strongly disagree	106 (33.3)	706 (27.4)	1.38 (1.06–1.79)*
**Has household been robbed in this neighborhood**			
Yes	145 (44.2)	1099 (42.6)	1.13 (0.89–1.44)
No	183 (45.8)	1479 (47.4)	1.00
**Neighborhood poverty**			
Not poor	155 (44.9)	980 (35.4)	1.00
Poor	92 (26.7)	915 (33.0)	0.70 (0.51–0.97)*
Very poor	98 (28.4)	874 (31.6)	0.62 (0.45–0.86)*

* p<0.05.

In multilevel models, both measures of neighborhood safety were associated with lifetime report of an asthma diagnosis, although a complex pattern emerged (Table 2). In Model 2, parents who reported their neighborhood as fairly safe or somewhat dangerous have a lower odds of reporting a child asthma diagnosis compared to those who reported their neighborhood as completely safe (OR 0.71; 95% CI 0.52–0.96 and OR 0.60; 95% CI 0.42–0.88 respectively). The effects of this measure of neighborhood safety were slightly attenuated but remained significant after controlling for additional family characteristics in Model 3 and assessment of the home environment in Model 4. In Model 2, parents who reported they could not trust their neighbors to keep their children safe was associated with a nearly 40% increase in lifetime asthma diagnosis compared to those who reported they could trust their neighbors to keep their children safe, after controlling for age, gender, and race/ethnicity (OR 1.39; 95% CI 1.07–1.81). This association remained essentially unchanged after accounting 194 for primary caregiver characteristics in Model 3, and the fully adjusted model which included the home and neighborhood environment in Model 4. Gender, age, and racial/ethnic differences persisted after accounting for primary caregiver and household characteristics. Primary caregiver’s history of asthma continued to have the strongest association with child’s report of lifetime asthma diagnosis (OR 2.82; 95% CI 1.97–4.03) while the effect of current smoking was attenuated. This suggests interplay between individual, household, and neighborhood factors and childhood asthma.

**Table 2:** Odds ratios (95% confidence intervals) for two-level random intercept models for reporting lifetime asthma exposure for exposure and covariates.

	Model 1^a^	Model 2^b^	Model 3^c^	Model 4^d^
*Neighborhood characteristics*				
**How safe to walk alone after dark in this neighborhood**				
Completely safe	1.00	1.00	1.00	1.00
Fairly safe	0.71 (0.53–0.97)*	0.71 (0.52–0.96)*	0.73 (0.53–0.99)*	0.72 (0.52–0.98)*
Somewhat dangerous	0.58 (0.41–0.84)*	0.60 (0.42–0.88)*	0.67 (0.46–0.98)*	0.67 (0.45–0.99)*
Extremely dangerous	0.77 (0.43–1.37)	0.69 (0.38–1.25)	0.69 (0.38–1.27)	0.65 (0.34–1.21)
**Adults watch out that kids are safe**				
Strongly agree or agree	1.00	1.00	1.00	1.00
Unsure, disagree, or strongly disagree	1.38 (1.06–1.79)*	1.39 (1.07–1.81)*	1.38 (1.05–1.81)*	1.40 (1.07–1.84)*
**Has household been robbed in this neighborhood**				
Yes	1.00	1.00	1.00	1.00
No	1.13 (0.89–1.44)	1.13 (0.88–1.44)	1.08 (0.84–1.38)	1.10 (0.86–1.42)
*Individual characteristics*				
**Gender**				
Male		1.00	1.00	1.00
Female		0.72 (0.57–0.91)*	0.70 (0.55–0.89)*	0.71 (0.56–0.91)*
**Age (years)**				
≤5		1.00	1.00	1.00
6–10		1.42 (1.04–1.94)*	1.43 (1.04–1.97)*	1.45 (1.05–1.99)*
11–14		1.97 (1.43–2.71)*	2.04 (1.47–2.82)*	2.03 (1.46–2.82)*
≥15		1.66 (1.15–2.40)*	1.51 (1.03–2.21)*	1.53 (1.04–2.25)*
**Race/ethnicity**				
Non-Hispanic White		1.00	1.00	1.00
Hispanic		0.95 (0.67–1.34)	1.40 (0.96–2.05)	1.37 (0.92–2.04)
Non-Hispanic Black		2.53 (1.65–3.89)*	2.29 (1.48–3.55)*	2.21 (1.41–3.49)*
Asian/other		1.33 (0.85–2.08)	1.35 (0.86–2.12)	1.30 (0.83–2.05)
*Family characteristics*				
**Primary caregiver’s asthma history**				
No prior asthma diagnosis			1.00	1.00
Prior asthma diagnosis			2.92 (2.04–4.16)*	2.82 (1.97–4.03)*
**Primary caregiver’s smoking status**				
Non-smoker			1.00	1.00
Smoker			1.26 (0.90–1.76)	1.30 (0.93–1.82)
**Health insurance during past 12 months**				
Insured			1.00	1.00
Partially insured or uninsured			1.11 (0.79–1.54)	0.78 (0.57–1.07)
**Primary caregiver’s education (years)**				
<12			1.00	1.00
12			1.62 (1.13–2.32)*	1.63 (1.13–2.35)*
>12			1.77 (1.25–2.51)*	1.78 (1.25–2.54)*
*Home characteristics*				
**Environment outside home unsafe for children**				
No				1.00
Yes				1.40 (0.91–2.16)
**Environment inside home unsafe for children**				
No				1.00
Yes				0.58 (0.30–1.11)
**Inside of home is crowded**				
No				1.00
Yes				1.03 (0.70–1.52)
**Visible rooms are neat and uncluttered**				
Yes				1.00
No				1.22 (0.93–1.60)
**Visible rooms are clean**				
Yes				1.00
No				1.38 (0.91–2.11)
*Neighborhood characteristics*				
**Neighborhood poverty**				
Not poor				1.00
Poor				0.85 (0.58–1.23)
Very poor				0.97 (0.63–1.47)

a Model 1 Unadjusted results of neighborhood characteristics.

b Model 2 Neighborhood characteristics adjusted for individual characteristics.

c Model 3 Neighborhood characteristics adjusted for individual and family characteristics.

d Model 4 Neighborhood characteristics adjusted for individual, family, and neighborhood characteristics.

## Discussion

The study demonstrated a complex pattern between various measures of neighborhood safety and asthma, which were robust after accounting for important covariates at the individual level (child’s age, gender, and race/ethnicity), family level (primary caregiver’s education, smoking status, asthma history, and public assistance), and household level (assessment of the home environment). These findings contribute to a growing body of evidence linking neighborhood violence to asthma outcomes and suggest that these relationships may operate differently in various contexts. Strengths of the study include the use of multilevel modeling to partially account for unmeasured confounding at the neighborhood level, as well as the diversity of the sample in regards to racial/ethnic and socioeconomic differences. However, these results should not be interpreted without also considering the limitations of the study.

First, our results indicated that primary caregiver’s with higher levels of education reported increased odds of asthma diagnosis, which could be a result of under-reporting among households of lower socioeconomic status due to access to health care. However, after controlling for child’s health insurance status, the positive association between parental education and child’s asthma diagnosis persisted. An additional limitation lies in the use of parental surrogate report of the child’s asthma, rather than review of medical records of additional measurements of lung. Additional measurements of asthma including date of diagnosis and measures of lung function have been added to Wave 2 of L.A.FANS, which will assist in reducing potential recall bias by parents and misclassification. While our study captured certain perceptions of neighborhood safety and experiences of neighborhood crime, we were unable to include additional measures of neighborhood safety such as police reports. However, previous studies have found strong correlations between perceptions of neighborhood safety and independent measures of crime. [31] In addition, parents’ perception could have captured additional information not provided by independent crime measures, since they reflect how parents may subsequently behave as a result of neighborhood beliefs. [3] However, we were unable to include more severe experiences of neighborhood crime, such as witnessing violent acts which have been examined in other contexts. [11], [32] Although our study did not control for outdoor environmental exposures, a study of Los Angeles County by Wilhelm and colleagues found that air pollution measures were only weakly correlated (r  = 0.3 or less) with social features of the environment such as neighborhood safety or social cohesion. [24] These findings suggest a complex geography of air pollution measures that may not fully explain the observed variation in asthma outcomes in Los Angeles.

Although data were unavailable to control specific indoor allergens, we controlled for assessments of the indoor environment that were available such as crowding, cleanliness, and presence of hazards within the home. Additional assessments of the home environment will be conducted using Wave 2 of L.A.FANS and will be included in future studies. Lastly due to the cross-sectional nature of the study design; we are unable to establish that the potential associations would be causal. A follow up of households within L.A.FANS (Wave 2) which includes additional measures of neighborhoods has been conducted; future analyses can be conducted to test the associations with prospective data.

While these findings are limited to Los Angeles and may not be generalized outside of this area, this study contributes to the growing body of literature linking neighborhood safety and childhood asthma, as few studies have examined this association outside of Northeastern cities. Although this study provides some evidence that unsafe neighborhoods are associated with increased asthma risk, findings suggest that the relationships between neighborhood social and environmental characteristics may be more complex in Los Angeles, prompting additional research in diverse contexts. As Los Angeles is distinct in regards to geography, racial/ethnic composition, and segregation patterns, it provides a unique an important context within which to study neighborhood effects on children’s health. Future studies of the interplay between the social environment and asthma will enable us to compare and contrast the experiences of children living in these neighborhoods to better studied cities, as well as continue to test our knowledge of neighborhood effects on health in a new and diverse context.
